# High-throughput quantification of protein structural change reveals potential mechanisms of temperature adaptation in *Mytilus* mussels

**DOI:** 10.1186/s12862-020-1593-y

**Published:** 2020-02-13

**Authors:** Ying-Chen Chao, Melanie Merritt, Devin Schaefferkoetter, Tyler G. Evans

**Affiliations:** Department of Biological Sciences, California State University East Bay, Hayward, CA 94542 USA

**Keywords:** Adaptation, Amino acid, Climate change, Hydrogen bond, Marine, *Mytilus*, Mussel, Protein, Salt bridge, Temperature

## Abstract

**Background:**

Temperature exerts a strong influence on protein evolution: species living in thermally distinct environments often exhibit adaptive differences in protein structure and function. However, previous research on protein temperature adaptation has focused on small numbers of proteins and on proteins adapted to extreme temperatures. Consequently, less is known about the types and quantity of evolutionary change that occurs to proteins when organisms adapt to small shifts in environmental temperature. In this study, these uncertainties were addressed by developing software that enabled comparison of structural changes associated with temperature adaptation (hydrogen bonding, salt bridge formation, and amino acid use) among large numbers of proteins from warm- and cold-adapted species of marine mussels, *Mytilus galloprovincialis* and *Mytilus trossulus*, respectively.

**Results:**

Small differences in habitat temperature that characterize the evolutionary history of *Mytilus* mussels were sufficient to cause protein structural changes consistent with temperature adaptation. Hydrogen bonds and salt bridges that increase stability and protect against heat-induced denaturation were more abundant in proteins from warm-adapted *M. galloprovincialis* compared with proteins from cold-adapted *M. trossulus*. These structural changes were related to deviations in the use of polar and charged amino acids that facilitate formation of hydrogen bonds and salt bridges within proteins, respectively. Enzymes, in particular those within antioxidant and cell death pathways, were over-represented among proteins with the most hydrogen bonds and salt bridges in warm-adapted *M. galloprovincialis*. Unlike extremophile proteins, temperature adaptation in *Mytilus* proteins did not involve substantial changes in the number of hydrophobic or large volume amino acids, nor in the content of glycine or proline.

**Conclusions:**

Small shifts in organism temperature tolerance, such as that needed to cope with climate warming, may result from structural and functional changes to a small percentage of the proteome. Proteins in which function is dependent on large conformational change, notably enzymes, may be particularly sensitive to temperature perturbation and represent foci for natural selection. Protein temperature adaptation can occur through different types and frequencies of structural change, and adaptive mechanisms used to cope with small shifts in habitat temperature appear different from mechanisms used to retain protein function at temperature extremes.

## Background

Temperature variation, including that resulting from anthropogenic climate change, is a pervasive feature of the biosphere that affects organism fitness through an influence on the three-dimensional structure of proteins [1]. Given that proteins are necessary for the growth, survival, and reproduction of most life on Earth, natural selection should favor amino acid sequences that optimize protein function in particular thermal environments [2–5]. Consistent with this hypothesis, clear patterns of adaptive variation have been discovered in the structural and functional properties of proteins from organisms adapted to different temperatures [5–9]. However, research to date is limited by two issues that have prevented insight into broader-scale patterns of temperature adaptation at the protein level. Firstly, previous work has focused on a small number of proteins, with most studies comparing thermal parameters in a single orthologue between species adapted to different temperatures [10–12]. Because the genomes of multicellular organisms encode thousands of proteins, little inference can be made from existing studies with regard to the proportion of proteins that must be evolutionarily modified in order to increase or decrease organism-level thermal tolerance. Secondly, in instances where the thermal properties of a larger number of proteins have been compared, study systems are typically extremophiles that exhibit very large differences in temperature tolerance and whose proteins are adapted to temperatures outside those encountered by the majority of life [7, 13–19]. Trends resolved in extremophile proteins may not be conserved in multicellular organisms and may not represent mechanisms underlying small adjustments in organism thermal tolerance, for example the few degrees Celsius increase that may be required for persistence in near-future climates [20]. Thus, there exists an opportunity to advance understanding of protein evolution by comparing thermal properties of proteins more comprehensively across the proteome, and doing so in closely related species that differ in thermal tolerance by a small margin.

A fundamental property of proteins is marginal stability: proteins are under selection to maintain a balance between stabilization, which prevents unfolding and aggregation; and flexibility, to allow the shape change necessary for catalysis, substrate binding, or protein-protein interactions [1, 21]. Temperature has a strong influence on the balance between stability and flexibility in proteins. The requirement that proteins have sufficient structural flexibility to undergo conformational change at a given temperature renders proteins susceptible to unfolding when temperature is increased and molecular interactions responsible for stabilizing proteins weaken. Conversely, decreases from optimal temperature cause mobile regions of proteins to become rigid, making shape changes necessary for function more difficult [5, 22–24]. This trade-off between stability and flexibility ensures no single protein can effectively function over the entire range of temperatures found across the biosphere [5, 25]. Alternatively, evolutionary processes fine-tune protein structures such that function is optimized at temperatures most frequently encountered by cells: orthologous proteins exhibit differences in thermal stability such that an ortholog from a cold-adapted species is more thermally labile than an ortholog from a warm-adapted species [3, 26–28].

Comparison of proteins adapted to different temperatures demonstrates that there are a multitude of ways in which thermal performance can be adaptively modified [29–31]. Altering hydrophobicity, charge, non-covalent interactions, volume, and cooperativity have all been implicated in protein temperature adaptation [14, 25, 32–36]. Also apparent from these comparisons is that the location of adaptive change within the folded protein is important. Natural selection can preferentially act upon particular regions during protein temperature adaption, such as the hydrophobic core or solvent-exposed surface, or target specific domains, such as coils, loops, helixes, or substrate binding sites, to adaptively shift thermal stability [5, 36, 37]. Often these mechanisms of thermal adaptation are reflected in specific patterns of amino acid use within proteins [6, 7, 9, 37]. Amino acid substitutions that adaptively vary the number of non-covalent hydrogen bonds and salt-bridges in proteins, including addition or removal of polar and charged residues, are a common means of optimizing protein function at given environmental temperatures [6]. The number of hydrogen bonds and salt bridges tends to increase in heat-tolerant (thermophilic) proteins as a means of protecting against denaturation at high temperatures [32, 33, 38–40]. Conversely, these stabilizing features tend to decrease in cold-tolerant (psychrophilic) proteins so that proteins remain sufficiently flexible to undergo conformational change at low temperatures [41–43]. Thermal stability is also modulated by the presence of hydrophobic and large volume amino acids. Residues with these chemical properties enhance thermal stability by restricting solvent access to the protein core [35], and accordingly, are frequently found in greater abundance in proteins that retain function in the heat [6, 34]. The ability of cysteine residues to form disulfide bonds, the rigidity of proline compared with other amino acids, and the lack of a side chain on glycine, also results in content of these amino acids often being correlated with protein structural stability and organism temperature tolerance [4].

In this study, software was developed to quantify hydrogen bonds, salt bridges, and amino acids across the proteomes of two closely related species of marine mussel that differ in temperature tolerance by a few degrees Celsius: *Mytilus galloprovincialis* and *Mytilus trossulus* [44]. This methodological innovation enabled comparison of structural features among large numbers of *Mytilus* proteins, and in turn, broader inference into how small shifts in environmental temperature influence the structural properties of proteins. *Mytilus* mussels have proven excellent model systems to examine protein temperature adaptation [44–46]. Although closely related, *M. galloprovincialis* and *M. trossulus* evolved in distinct thermal environments. *M. trossulus* and *M. galloprovincialis* are members of the ‘blue mussel complex’, which also includes *M. edulis* [47]. *M. trossulus*, a native of the cold Northeast Pacific Ocean, appears to be the ancestral species of the complex, with divergence into *M. edulis* occurring as a result of migration through a transient opening in the Bering Strait and into the Atlantic Ocean approximately 3.5 million years ago [48]. Subsequently, *M. edulis* expanded its range throughout the North Atlantic and into the warmer waters of the Mediterranean Sea, where populations became isolated and gave rise to *M. galloprovincialis* about 2 million years ago [49]. Differences in prevailing temperatures experienced during evolutionary history are reflected in the thermal tolerances of *M. galloprovincialis* and *M. trossulus*: the upper critical temperature of *M. galloprovincialis* is approximately 4 °C higher than that of *M. trossulus* as a result of evolution in a comparatively warmer environment (LT_50_ = 38 °C and LT_50_ = 34 °C, respectively) [12]. Physiological performance also varies between these species in a manner that is consistent with adaptation to different temperatures. All physiological (e.g. heart rate [50]), biochemical (e.g. enzyme activities [44–46]), and molecular (e.g. gene expression [51, 52];) comparisons made to date show *M. galloprovincialis* to be more tolerant of heat and *M. trossulus* to be more tolerant of cold.

Orthologous enzymes from *M. galloprovincialis* and *M. trossulus* exhibit differences in structural stability and function that are congruent with their thermal tolerances and with known mechanisms of temperature adaptation. Cytosolic malate dehydrogenase (cMDH) and isocitrate dehydrogenase (IDH) from warm-adapted *M. galloprovincialis* sustain activity and optimal substrate binding at higher temperatures than orthologs from cold-adapted *M. trossulus* [45, 46]. Enhanced function of *M. galloprovincialis* cMDH and IDH results from amino acid substitutions that increase the number of hydrogen bonds in order to protect regions undergoing conformational change from unfolding at high temperatures [3, 45, 46]. However, the present state of research provides an incomplete perspective about how natural selection modifies *Mytilus* proteins to optimize function in their respective temperature regimes. Only a small number of *Mytilus* proteins have been analyzed: the influence of temperature on protein function has been investigated in only six *Mytilus* orthologs to date [45, 46]. Thermal stability has been predicted in silico for large numbers of *Mytilus* proteins, and importantly, this work has confirmed the presence of more stabilizing amino acids in the proteins of *M. galloprovincialis* compared with proteins from *M. trossulus* [53]. However, the methodology used in this previous study did not allow for inference into the potential contributions of hydrogen bonds or salt bridges to variation in protein thermal stability, nor could it identify the specific types of amino acid substitution responsible for differences in protein thermal stability between *Mytilus* congeners.

Here, individual amino acids, hydrogen bonds, and salt bridges were counted in large numbers of *M. galloprovincialis* and *M. trossulus* proteins in order to address several unresolved questions surrounding protein evolution in *Mytilus* and protein temperature adaptation in general [1, 5, 54]. First, are small differences in environmental temperature sufficient to cause changes in amino acid use and non-covalent interactions that are consistent with mechanisms of protein temperature adaptation? Second, what proportion of the proteome needs to be adaptively modified to alter organism temperature tolerance by a small margin? Third, are proteins with particular functions more likely than others to be acted on by natural selection during temperature adaptation? Finally, are certain mechanisms of adaptive change favored among *Mytilus* proteins and do these changes parallel those occurring in proteins adapted to temperature extremes? Answers to these questions have broad implications, from improving estimates of the capacity to cope with climate change, to better understanding the process of protein evolution over the range of temperatures found throughout the biosphere.

## Methods

### Amino acid sequences, protein modeling, and quantifying hydrogen bonds and salt bridges

Hydrogen bonds or salt bridges are rarely directly measured within proteins [55]. Instead, these structural features are predicted based on the spatial arrangement of atoms within three-dimensional protein models created from amino acid sequences. *M. galloprovincialis* and *M. trossulus* protein sequences were obtained by searching for the keyword “*Mytilus*” in the UniProt database [56] and then selecting all sequences for each species using the “popular organisms” tab on the results page. Resulting sequences were downloaded in .fasta format and used to create models of each protein using SWISS MODEL [57, 58]. SWISS MODEL software uses sequence information to search a database of proteins for which the three-dimensional structure has been solved using X-ray crystallography, nuclear magnetic resonance, or electron microscopy techniques [59]. When an appropriate template has been identified, the software develops a model of the protein of interest, storing information about the location of each amino acid in a protein database (.pdb) file. SWISS MODEL will model proteins in the macromolecular assembly that has either been shown to be or is believed to be the functional form of the molecule. SWISS MODEL templates are annotated with information about the whether the protein functions as a monomer or an oligomer (referred to as the biological assembly or biounit), and this quaternary structure annotation is used to model the target sequence in its functional form [58]. SWISS MODEL will include a ligand in protein models if the highest scoring template includes a biologically relevant ligand and amino acid residues involved in ligand binding are conserved in the target–template sequence alignment [58].

*Mytilus* .pdb files generated by SWISS MODEL were uploaded to a web server of the HBOND program that uses the spatial arrangement of amino acids recorded in the .pdb file to predict hydrogen bonding [60]. The HBOND algorithm predicts hydrogen bond formation at a maximum donor–acceptor distance of 3.5 Å and maximum hydrogen–acceptor distance of 2.5 Å. HBOND does not consider bond angle in its prediction criteria. HBOND will calculate hydrogen bonds between a protein and ligand if a ligand is included in the model, but does not consider hydrogen bonds that could form between the protein and solvent [60]. The same .pdb files were also uploaded to a different web server called Evaluating Salt BRIdges in proteins (ESBRI) in order to predict salt bridges in each protein [61]. The ESBRI algorithm predicts formation of a salt bridge if at least one aspartate or glutamate side-chain carboxyl oxygen atom and one side-chain nitrogen atom of arginine, lysine, or histidine are within a distance of 4.0 Å. Predicted hydrogen bonds and salt bridges were then counted from the HBOND and ESBRI output files, respectively, to get the total number of each feature in each *Mytilus* protein of interest.

### Software development

Manual completion of the protocol described above constrains the number of proteins that can be assayed. To allow high-throughput quantification of hydrogen bonds and salt bridges in proteins of interest, custom software was developed that automated the data acquisition pipeline. The software required as input a .fasta file containing all proteins of interest. Individual sequences from the .fasta file were then sequentially passed to SWISS MODEL in order to generate protein models. Resulting .pdb files were then automatically uploaded to HBOND and ESBRI servers in order to predict hydrogen bonds and salt bridges. Pertinent information was extracted from output files at each step of the process, including the unique UniProt ID and length of each protein (extracted from the .fasta file), QMEAN (Qualitative Model Energy ANalysis, a quality control metric extracted from the .pdb file), and the total number of hydrogen bonds and salt bridges (extracted from the HBOND and ESBRI output files). These parameters were saved to a database until data from all proteins in the .fasta input file had been processed by the software.

### Quality control and statistics

QMEAN is a score generated by SWISS MODEL to estimate the quality of protein models. QMEAN is a combined scoring function that incorporates five different structural descriptors to determine the major geometrical aspects of protein structure, with a higher score indicative of greater agreement between the protein model and the native conformation of the template protein [62]. QMEAN score was used to remove low quality models of *Mytilus* proteins that could cause inaccurate prediction of hydrogen bonds and salt bridges. Following SWISS MODEL recommendations, protein models with QMEAN scores less than − 5 were removed from analyses [63]; personal communication, Andrew Waterhouse, Swiss Institute of Bioinformatics]. Duplicate proteins within each species were removed by retaining the single protein model with the highest QMEAN score. This filtering step was necessary because the UniProt database may contain multiple entries for the same protein (i.e. proteins annotated exactly the same, but assigned a different UniProt ID) and because SWISS MODEL often generates multiple models for the same protein using different templates. Proteins annotated as “uncharacterized protein” may not represent unique proteins and were removed from analyses. To control for differences in length when comparing *Mytilus* proteins (long proteins composed of many amino acids would be expected to have more hydrogen bonds and salt bridges than short proteins), the number of hydrogen bonds and the number of salt bridges were each divided by the total number of amino acids in each protein. The resulting values, expressed as the number of hydrogen bonds or salt bridges per amino acid for each unique protein, were then used in statistical tests comparing sets of proteins between the two mussel species. Proteins annotated exactly the same between each species were considered orthologs.

Anderson-Darling, Ryan-Joiner, and Kolmogorov-Smirnov tests of normality all indicated that the number of hydrogen bonds per amino acid and the number of salt bridges per amino acid in *Mytilus* proteins were not normally distributed. Standard methods of data transformation (e.g. log, square, and Box-Cox transformations) did not correct this trend. Given these results, non-parametric Mann-Whitney U tests were used to determine whether the median number of hydrogen bonds or salt bridges per amino acid significantly differed between *M. galloprovincialis* and *M. trossulus*. Statistical tests were performed using MiniTab version 18.0 with *p*-values less than 0.05 considered significant. Box plots and frequency histograms used to illustrate hydrogen bond and salt bridge data were created using Microsoft Excel 2016.

### Over-representation of protein ontologies

Over-representation analysis was used to determine if *Mytilus* proteins with particular functions were more likely than others to have modified the number of hydrogen bonds or salt bridges. Over-representation analysis is a statistical approach that uses functional information to identify categories of proteins (called ontologies [64]) found in a greater proportion than expected within a user-defined list. Significance is determined by the probability that the number of proteins from a given ontology in the user-defined list occurred by chance relative to the number of proteins from this same ontology in a larger background list. Over-representation analysis was performed using the R package TopGO using the “classic” algorithm in which each ontology category from the biological process, molecular function, and cellular component databases are tested independently [65]. Background lists were created for each species by including all unique proteins with QMEAN scores greater than − 5. Ontologies with unadjusted Fisher Exact test *p*-values less than 0.05 were considered significantly over-represented (correction for multiple testing can produce misleading results and is not recommended in TopGO [66]).

### Amino acid use

Custom software used to quantify hydrogen bonds and salt bridges was also programmed to count the number of each amino acid in each protein from the input .fasta file. Chi-square tests of independence were then used to determine if the proportions of each amino acid differed among sets of *M. galloprovincialis* and *M. trossulus* proteins, with *p*-values less than 0.05 considered significant [67]. This statistical test generates observed and expected values for each amino acid for each species; thus, differences in these two values provide insight into amino acid use and potential mechanisms of temperature adaptation among *Mytilus* proteins. Observed values represent total counts of each amino acid within the proteins from each species. Expected values are calculated by determining the frequency of each amino acid among proteins from both species in a given protein set and then multiplying this frequency by the actual observed count of each amino acid in the proteins from each species individually in the set. Amino acid use was first analyzed in subsets of proteins with the greatest number of hydrogen bonds and salt bridges per amino acid so that specific amino acid substitution patterns could be related to differences in the number of these structural features between *M. galloprovincialis* and *M. trossulus*. Amino acid content of all unique proteins with sequence information for each species (including protein sequences from which SWISS-MODEL could not create a high quality model and therefore hydrogen bonds and salt bridges could not be quantified) was also analyzed using the same statistical approach. Analyzing all proteins provided a broader overview of amino acid use among *Mytilus* proteins.

Amino acid data were also used to test the hypothesis that the proportion of hydrophobic and large volume amino acids would be greater among the proteins of warm-adapted *M. galloprovincialis* compared with the proteins of cold-adapted *M. trossulus*. This hypothesis was statistically evaluated by calculating Spearman rank order correlation coefficients (rho) between the deviation (i.e. observed-expected) of each amino acid in *M. galloprovincialis* relative to *M. trossulus* and hydrophobicity rank [68] or volume rank [69] for each residue. All unique proteins with amino acid sequence information from each species were used in this analysis. These same tests were performed on proteins with the greatest number of hydrogen bonds or salt bridges per amino acid to determine if changes in hydrophobicity or residue volume were occurring in the same subset of proteins exhibiting differences in hydrogen bonds and salt bridges between *Mytilus* species.

## Results

### Protein sequences, modeling, and quality control

The UniProt database contained 4609 protein sequences for *M. galloprovincialis* and 725 protein sequences for *M. trossulus* (Table 1). Three-dimensional modeling of these 5334 protein sequences resulted in 1325 models generated for *M. galloprovincialis* and 669 models for *M. trossulus*. An X in the input sequence (indicating a missing or ambiguous amino acid) or absence of a suitable template protein in the SWISS MODEL database caused the number of protein models generated to be smaller than the number of input sequences. Removal of models for duplicate proteins within each species (for example Cytochrome C oxidase 1 accounted for 137 and 228 models in *M. galloprovincialis* and *M. trossulus*, respectively), left 574 unique protein models in *M. galloprovincialis* and 85 unique protein models in *M. trossulus*. Of the remaining models, 482 and 58 had QMEAN scores greater than − 5 in *M. galloprovincialis* and *M. trossulus*, respectively, and were used in subsequent analyses of hydrogen bonding and salt bridge formation (Additional file 1: Figures S1 and S2). Within this set of high-quality models, 19 proteins were annotated exactly the same between *M. galloprovincialis* and *M. trossulus* and were presumed to be orthologs (Additional file 1: Figure S3).

**Table 1:** Summary of protein sequences, protein modeling, and filtering

	*M. galloprovincialis*	*M. trossulus*
Number of proteins in UniProt database	4609	725
Number of protein models generated in SWISS MODEL	1325	669
Number of models for unique proteins	574	85
Number of models for unique proteins with QMEAN > −5	482	58
Number of orthologous proteins	19	19

### Hydrogen bonds and salt bridges

**Fig. 1:**
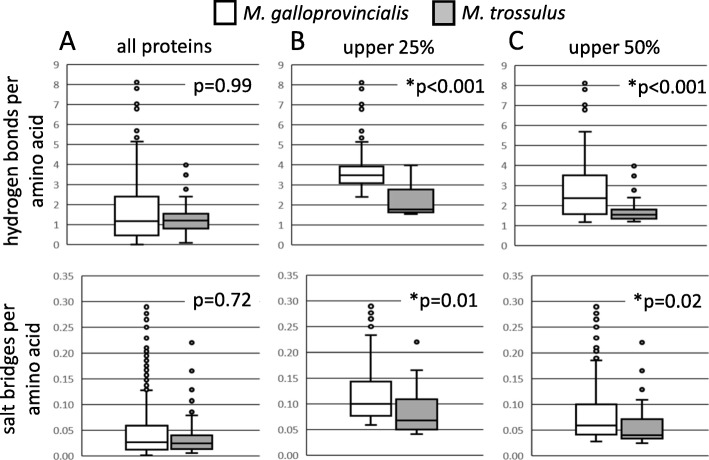
Box and whisker plots of hydrogen bonds and salt bridges per amino acid in *M. galloprovincialis* (white) and *M. trossulus* (grey) for (**a**) all unique proteins (**b**) the upper 25% of proteins with the most hydrogen bonds or salt bridges per amino acid, and (**c**) the upper 50% of proteins with the most hydrogen bonds or salt bridges per amino acid. Mann-Whitney U tests show that the upper 25 and 50% of proteins with the most hydrogen bonds or salt bridges per amino acid differs significantly between *M. galloprovincialis* and *M. trossulus* (asterisks, *p* < 0.05)

**Fig. 2:**
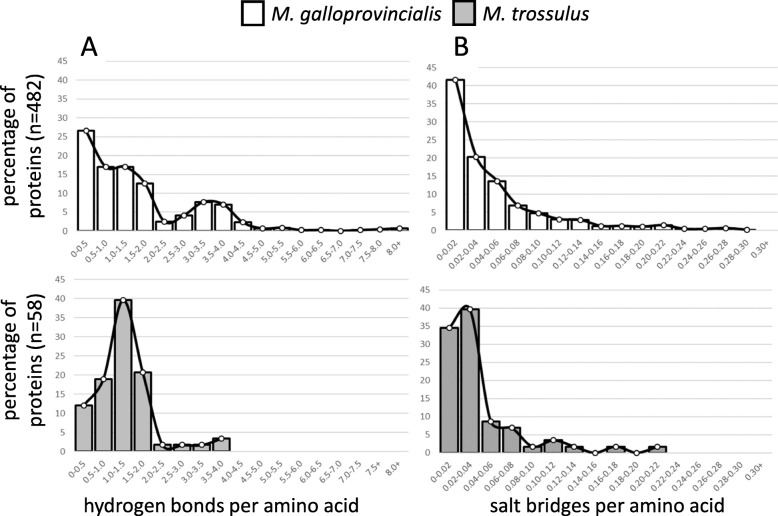
Frequency histograms showing the percentage of *M. galloprovincialis* (white) and *M. trossulus* (grey) proteins within particular ranges of (**a**) hydrogen bonds per amino acid and (**b**) salt bridges per amino acid

The number of hydrogen bonds and salt bridges per amino acid were compared to test the hypothesis that these stabilizing features are more common within the proteins of warm-adapted *M. galloprovincialis* than within the proteins of cold-adapted *M. trossulus.* The median number of hydrogen bonds or salt bridges per amino acid among all unique proteins did not differ significantly between *M. galloprovincialis* and *M. trossulus* (Fig. 1a). There was also no significant difference between the two species in the median number of hydrogen bonds or salt bridges per amino acid in the set of 19 orthologous proteins. Given these results, an alternative hypothesis is that enhanced heat tolerance in *M. galloprovincialis* is the result of added stability in a smaller subset of proteins [46]. This hypothesis was tested by generating frequency histograms that compare the percentage of proteins within different ranges of hydrogen bonds and salt bridges per amino acid between the two species (Fig. 2). These histograms show that *M. galloprovincialis* has a greater percentage of proteins within the upper range of hydrogen bonds and salt bridges per amino acid compared with *M. trossulus*. For example, 5.4% (26 of 482) of *M. galloprovincialis* proteins have more than 4.0 hydrogen bonds per amino acid, whereas none of the 58 *M. trossulus* proteins analyzed meet this criteria (Fig. 2a). Similarly, 6.6% (32 of 482) of *M. galloprovincialis* proteins have more than 0.14 salt bridges per amino acid compared with only 3.4% (2 of 58) of *M. trossulus* proteins (Fig. 2b).

Frequency histograms indicate that the number of hydrogen bonds and salt bridges differ in a subset of *Mytilus* proteins. This possibility was further evaluated by statistically comparing the median number of hydrogen bonds and salt bridges per amino acid in the upper quartiles and upper halves of proteins in each species. The upper 25% of *M. galloprovincialis* proteins (*n* = 121) contained significantly more hydrogen bonds (*p* < 0.001) and salt bridges (*p* = 0.011) per amino acid than did the upper 25% of *M. trossulus* proteins (*n* = 15) (Fig. 1b). This pattern also extended to the upper 50% of proteins, with *M. galloprovincialis* (*n* = 241) having significantly more hydrogen bonds (*p* < 0.001) and salt bridges (*p* = 0.024) per amino acid compared with *M. trossulus* (*n* = 29) (Fig. 1c).

### Over-representation of protein ontologies

Over-representation analysis was performed on the upper 25% and upper 50% of *M. galloprovincialis* proteins to determine if potentially adaptive increases in hydrogen bonding and salt bridge formation occurred disproportionately in proteins within certain pathways, in proteins with particular functions, or in proteins at specific cellular locations (Additional file 1: Figure S4). The upper 25% of *M. galloprovincialis* proteins with the greatest number of hydrogen bonds per amino acid contained a significantly greater proportion of proteins involved in carboxylic acid metabolism (e.g. “tricarboxylic acid cycle”), programmed cell death (e.g. “apoptotic process”), and antioxidant production (e.g. “glutathione transferase activity”) (Table 2). Proteins annotating to the carboxylic acid metabolism ontology included cMDH and IDH. Proteins annotating to cell death ontologies included regulatory molecules within the apoptosis signaling cascade (e.g. multiple isoforms of p63) and proteases involved in the destruction of proteins (e.g. caspase 3/7–1 and 3/7–2) [70–73]. Six isoforms of glutathione-S-transferase enzymes involved in the production of antioxidants were present in the upper 25% of *M. galloprovincialis* proteins with the most hydrogen bonds per amino acid [74]. Ontologies relating to these same three processes were also over-represented in the upper 50% of *M. galloprovincialis* proteins with the most hydrogen bonds per amino acid, including “oxidation-reduction process”(carboxylic acid metabolism), “cysteine-type peptidase activity” (cell death), and “transferase activity” (antioxidant production). Cellular component ontology terms were not significantly over-represented in either the upper 25% or upper 50% of *M. galloprovincialis* proteins with the greatest number of hydrogen bonds per amino acid (Additional file 1: Figure S4).

**Table 2:** Select ontology over-representation among the 25% of *M. galloprovincialis* proteins with most hydrogen bonds per amino acid

Gene ontology ID	Ontology	Number in background	Number in upper 25%	Number expected in upper 25%	*P*-value
A. Biological process					
GO:0006082	organic acid metabolic process	3	3	0.38	0.001
GO:0006091	carboxylic acid metabolic process	3	3	0.38	0.001
GO:0006099	tricarboxylic acid cycle	2	2	0.25	0.014
GO:0006915	apoptotic process	15	6	1.88	0.001
GO:0008219	cell death	15	6	1.88	0.001
B. Molecular function					
GO:0016740	transferase activity	10	7	1.44	< 0.001
GO:0004364	glutathione transferase activity	3	3	0.43	0.003

In general, the same ontologies that were significantly over-represented among sets of proteins with the most hydrogen bonds per amino acid were also over-represented among sets of proteins with the most salt bridges per amino acid, including those relating to carboxylic acid metabolism (e.g. “organic acid metabolic process”), cell death (e.g. “apoptotic process”), and antioxidant production (e.g. “glutathione transferase activity”) (Table 3; Additional file 1: Figure S4). Overlap between proteins with the most hydrogen bonds and those with the most salt bridges was not unexpected since salt bridges contain a hydrogen bond. Consistent with this assumption, 71% of proteins (*n* = 200) were found in both the list of the 50% of proteins with the greatest number of hydrogen bonds per amino acid and the list of the 50% of proteins with the greatest number of salt bridges per amino acid in *M. galloprovincialis*. Spearman’s rank tests show that the number of hydrogen bonds per amino acid was significantly positively correlated with the number of salt bridges per amino acid for all proteins, as well as the upper 50 and 25% of proteins with the most hydrogens bonds or salt bridges per amino acid for both species (Additional file 1: Figure S5).

**Table 3:** Select ontology over-representation among the 25% of *M. galloprovincialis* proteins with most salt bridges per amino acid

Gene ontology ID	Ontology	Number in background	Number in upper 25%	Number expected in upper 25%	*P*-value
A. Biological process					
GO:0006082	organic acid metabolic process	3	3	0.83	0.019
GO:0019752	carboxylic acid metabolic process	3	3	0.83	0.019
GO:0006915	apoptotic process	15	8	4.17	0.018
GO:0008219	cell death	15	8	4.17	0.018
B. Molecular function					
GO:0016740	transferase activity	10	8	2.80	< 0.001
GO:0004364	glutathione transferase activity	3	3	0.84	0.020

### Amino acid use

Amino acids differ in their ability to form hydrogen bonds and salt bridges, thus certain amino acids were expected to be more frequent in *M. galloprovincialis* proteins that exhibited higher rates of hydrogen bonding and salt bridge formation compared with *M. trossulus* (Additional file 1: Figures S6 and S7). A chi square test for independence demonstrates that amino acid use was significantly different between *M. galloprovincialis* and *M. trossulus* in both the upper 25% and upper 50% of proteins with the greatest number of hydrogen bonds per amino acid. Amino acids with hydroxyl (serine and threonine), amide (asparagine and glutamine), aromatic (histidine, tyrosine, tryptophan), or charged groups (aspartate, glutamate, arginine, lysine), are most likely to form conventional (OH--O or OH--N) hydrogen bonds [75]. The upper 50% of *M. galloprovincialis* proteins with the most hydrogen bonds per amino acid contained 105 more of these 11 amino acids than expected compared with the upper 50% of *M. trossulus* proteins (1.4% higher use rate; Table 4). Similarly, the upper 25% of *M. galloprovincialis* proteins contained 51 more of these 11 amino acids than expected compared with the upper 25% of *M. trossulus* proteins (1.2% higher use rate; Additional file 1: Figure S8). These trends were caused by increases in the number of aspartate, asparagine, glutamine, and threonine residues in both the upper 25 and 50% of *M. galloprovincialis* proteins. Amino acid use did not differ significantly between *M. galloprovincialis* and *M. trossulus* among the set of 19 presumed orthologs (Additional file 1: Figure S8).

**Table 4:**
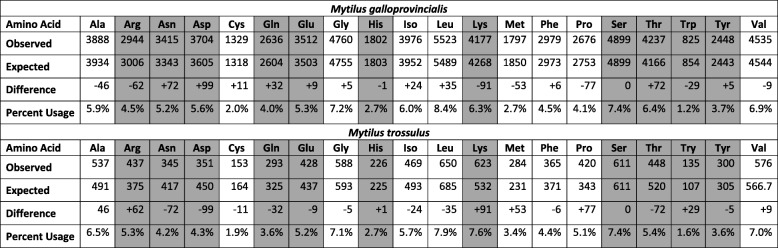
Amino acid use among the 50% of *Mytilus* proteins with the most hydrogen bonds per amino acid. Amino acids most likely to form conventional hydrogen bonds are shaded grey [75]

Salt bridges form as a result of attraction between oppositely charged residues; thus, the proportions of amino acids with charged side chains (arginine, aspartate, lysine, and glutamate) were predicted to differ between the proteins of *M. galloprovincialis* and *M. trossulus* (Additional file 1: Figures S6 and S7). The upper 25% and upper 50% of proteins with the greatest number of salt bridges per amino acid differed significantly in amino acid composition between *Mytilus* species (Additional file 1: Figure S8). Among the 50% of proteins with the most salt bridges per amino acid, there were 80 more negatively charged residues than expected in *M. galloprovincialis* compared with *M. trossulus*, a trend caused by an increase in the number of aspartate residues (1.2% higher use rate; Table 5). The number of positively charged amino acids decreased in *M. galloprovincialis* proteins relative to *M. trossulus*. The 50% of *M. galloprovincialis* proteins with the most salt bridges per amino acid had 132 fewer lysine residues (2.0% lower use rate) and 100 fewer arginine residues than expected (1.5% lower use rate) compared with *M. trossulus*. Inclusion of histidine, which is positively charged at physiological pH, has little effect on these trends because the number of histidine residues had very small deviations from the expected number between species (Table 5).

**Table 5:**
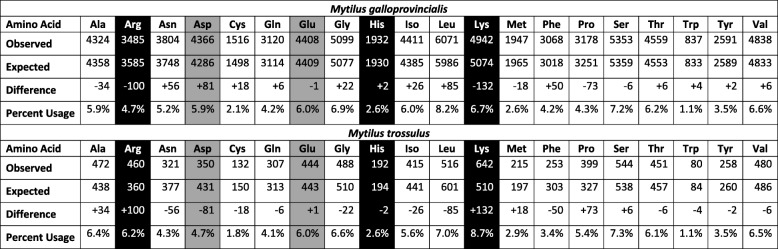
Amino acid use among the 50% of *Mytilus* proteins with the most salt bridges per amino acid. Positively charged residues are shaded black. Negatively charged residues are shaded grey

**Fig. 3:**
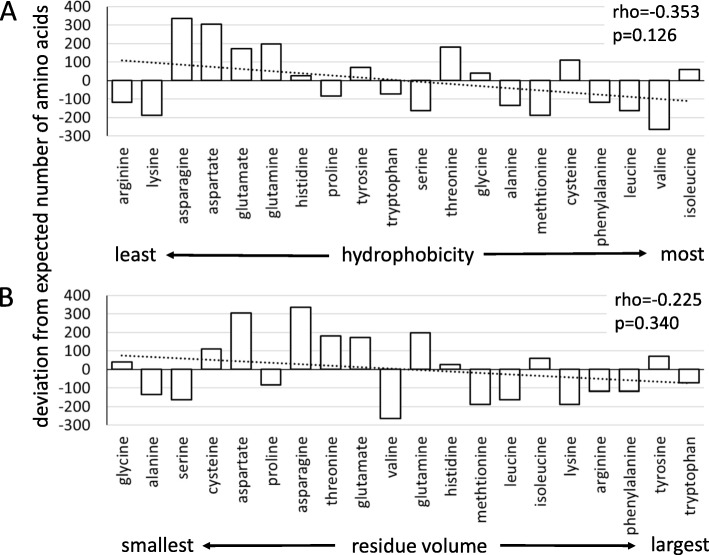
Spearman rank order correlation between the deviation in amino acid use in *M. galloprovincialis* relative to *M. trossulus* for all proteins and (**a**) rank residue hydrophobicity or (**b**) rank residue volume

Analysis of thermophilic proteins from prokaryotes indicates that stability and function in the heat can be improved by increasing the number of disulfide bridges, by reducing glycine content, by increasing hydrophobicity of the protein core, and by increasing protein volume [1, 6, 76]. Deviations in amino acid use were used to determine whether these same mechanisms of protein temperature adaptation contribute to protein evolution in *Mytilus* mussels. Amino acid use provides support for some, but not all, of these changes occurring in *Mytilus* proteins (Additional file 1: Figure S8). The complete set of *M. galloprovincialis* proteins had more cysteines than expected compared with all *M. trossulus* proteins (+ 109; 0.6% higher in use rate), similar to the amino acid composition of thermophilic proteins. However, glycine content increased in warm-adapted *M. galloprovincialis* relative to *M. trossulus* (+ 39; 0.2% higher use rate), a result that does not conform to that of thermophilic proteins. Also in opposition to patterns observed in thermophiles, *M. galloprovincialis* did not increase the proportion of hydrophobic or large volume amino acids relative to *M. trossulus* when all proteins are considered (Fig. 3). There is also no significant positive correlation between the proportion of hydrophobic or large volume amino acids in the upper 25% or upper 50% of *Mytilus* proteins with either the highest number of hydrogen bonds per amino acid or the highest number of salt bridges per amino acid (Additional file 1: Figure S9).

## Discussion

The study of protein evolution presently benefits from a vast amount of sequence data, but a relatively small body of structural data [24]. Software developed and applied here addresses this deficiency by capitalizing on existing amino acid sequence information to generate new protein structural data. Liao et al. (2017) emphasize that effects of a single substitution cannot be fully interpreted by examining only localized bonding in specific amino acid residues, but must include a broader analysis of changes in protein structure [10]. Here, for the first time, the total number of hydrogen bonds, salt bridges, and amino acids in large numbers of *Mytilus* proteins were analyzed to reveal broader patterns of structural change between species. Fields et al. (2015) iterate two unresolved issues regarding protein temperature adaptation [5]. One, that we are currently unable to place comparative studies of protein evolution into the broader context of the proteome because too few proteins have been analyzed. And two, the need to identify protein classes or biochemical pathways that are more frequently acted upon by natural selection during temperature adaptation. Analyses performed here suggest that comprehensive modification of the proteome is not required to alter thermal tolerance by a small margin because only a small percentage of *Mytilus* proteins showed structural variation between species consistent with temperature adaptation. Over-representation analysis indicated that enzymes, which require large conformation change during catalysis, were disproportionately acted on by natural selection during protein temperature adaptation in *Mytilus*. Finally, it remains important to evaluate whether a given evolutionary solution for sustaining protein function at different temperatures is conserved across phylogenetic boundaries [1, 12, 77]. Structural changes associated with temperature adaptation in *Mytilus* mussels were contrasted with mechanisms established in extremophile proteins to address this issue.

### Variation in hydrogen bonding and salt bridge formation among *Mytilus* proteins

Moderately higher upper critical temperature of *M. galloprovincialis* compared with *M. trossulus* was associated with higher numbers of stabilizing hydrogen bonds and salt bridges in a small percentage of proteins. A broad interpretation of this result is that small increases in organism thermal tolerance may be achieved through evolutionary modification of only part of the proteome. Other analyses of *Mytilus* proteins substantiate this interpretation, demonstrating that structural or functional changes that accompany temperature adaptation occur in only a portion of the proteins analyzed. The effect of temperature on the kinetic properties of six *Mytilus* enzyme othologs have been assessed to date, and in only two, cMDH and IDH, did Michaelis-Menten (Km) values differ significantly between *M. galloprovincialis* and *M. trossulus* [45, 46]. Thermal sensitivities of the other four orthologs were remarkably similar between *Mytilus* species over a wide range of temperatures [46]. In silico estimates of stability differences among *Mytilus* proteins further corroborate that evolutionary processes underlying temperature adaptation in these species favor changes occurring in particular proteins rather than changes occurring widely across the proteome [53]. A significantly greater number of stabilizing amino acid substitutions occurred in the proteins of warm-adapted *M. galloprovincialis* compared with cold-adapted *M. trossulus*. However, this signal of thermal adaptation was caused by a small number of *M. galloprovincialis* outlier proteins in which amino acid changes resulted in large increases in protein stability. Importantly, removal of these outlier proteins from statistical models caused differences in average protein thermal stability between the two species to become insignificant [53]. Even very large differences in organism temperature tolerance do not appear to result from comprehensive modification of the proteome. Comparison of extremophile Archaea and bacteria adapted to temperatures that range from − 12 °C to 103 °C indicates that protein thermal stability is modified to different extents across the proteome. Stability was increased in many thermophile proteins as expected; however, other proteins exhibited no difference in thermal stability between thermophiles and psychrophiles, and some proteins exhibited greater stability in cold-tolerant species contrary to a priori predictions [19].

### Functional bias in *Mytilus* protein temperature adaptation

Significant differences in hydrogen bonding and salt bridge formation occurring among a subset of *Mytilus* proteins indicates that the strength of temperature selection varies across the proteomes of these mussels. A possible explanation for this trend is that selection is most intense on classes of proteins that are especially perturbed by temperature or particularly important for maintaining homeostasis during thermal stress. The requirement for large conformational change during catalysis may render enzymes more susceptible to thermal stress than classes of protein where function is dependent on smaller movements [11, 19, 46]. Variation in hydrogen bonding and salt bridge formation among *Mytilus* proteins provides support for this hypothesis. The set of *M. galloprovincialis* proteins with the greatest number of hydrogen bonds and salt bridges per amino acid contained a greater than expected proportion of enzymes. “Peptidase”, “oxidoreductase”, and “transferase”, were the top over-represented molecular function ontologies among the 50% of *M. galloprovincialis* proteins with the most hydrogen bonds per amino acid. cMDH and IDH are enzymes that exhibit temperature-dependent differences in catalytic activity between *Mytilus* species consistent with temperature adaptation [45, 46]. Both cMDH and IDH are large, multi-subunit proteins that undergo substantial conformational shifts during ligand binding [5, 46], and both were among the *M. galloprovincialis* proteins with the most hydrogen bonds and salt bridges per amino acid (Additional file 1: Figures S1 and S2). Enzymatic bias in selection is also considered a causative factor for inconsistent temperature adaptation among the proteins of extremophiles. Proteome comparison of 24 prokaryotic species adapted to a wide range of temperatures showed that proteins annotating to “enzyme regulation” and “catalytic activity” ontologies experienced the greatest shifts in thermal stability [19].

Enzymes functioning within biochemical pathways that are crucial to surviving periods of thermal stress may also be subject to strong selection pressure. Antioxidant systems and programmed cell death are components of the conserved cellular stress response that is induced following temperature increase and that works to maintain homeostasis under suboptimal thermal conditions [78, 79]. Enzymes from these two important processes were over-represented among the small group of proteins with significantly different numbers of hydrogen bonds and salt bridges between *Mytilus* species. Over-represented ontologies relating to carboxylic acid metabolism were caused by high numbers of hydrogen bonds and salt bridges in cMDH and IDH of *M. galloprovincialis.* Although isoforms of these two enzymes participate in the tricarboxylic acid cycle (hence annotation to this ontology), the specific isoforms analyzed here function outside this pathway, and alternatively support the hypothesis that differences in the capacity of *M. galloprovincialis* and *M. trossulus* to cope with oxidative stress contributes to their divergent heat tolerances [52]. Exposure to heat stress increases the production of damaging reactive oxygen species (ROS) through an effect on the electron transport chain used for ATP production in the mitochondria [80]. Heat-induced increases in ROS can be ameliorated by reducing influx of NADH through the electron transport chain or by increasing production of antioxidants that can scavenge these molecules [74]. Both cMDH and IDH can influence these defense mechanisms: cMDH influences the influx of NADH to the mitochondria through the malate-aspartate shuttle and IDH catalyzes production of NADPH that is needed by antioxidants like glutathione [74]. There is evidence to suggest that the higher heat tolerance of *M. galloprovincialis* compared with *M. trossulus* is due in part to an ability to control oxidative stress by enhancing the production of antioxidants rather than slowing the rate of NADH entry to the electron transport chain (and subsequently limiting ATP production) [52, 81]. Enhancing the thermal stability of enzymes like IDH and cMDH would allow chemical reactions that reduce oxidative stress and sustain ATP production to continue at higher temperatures in *M. galloprovincialis* compared with *M. trossulus*. This hypothesis is consistent with predicted increases in the total number of hydrogen bonds in *M. galloprovincialis* orthologs of IDH (*n* = 1646) and cMDH (*n* = 1326) compared with IDH (*n* = 1644) and cMDH (*n* = 1320) of *M. trossulus* (Additional file 1: Figures S1 and S2). Furthermore, other antioxidant enzymes were among the upper 25% of *M. galloprovincialis* proteins with the most hydrogen bonds and salt bridges per amino acid, including superoxide dismutase and glutathione S-transferase, suggesting that these enzymes may also be adapted to retain function at higher temperatures in *M. galloprovincialis* than in *M. trossulus*.

Programmed cell death (apoptosis) can be used to eliminate dysfunctional cells when macromolecular damage from thermal stress exceeds a cell’s capacity for repair [78, 79]. Apoptosis is an important aspect of the response to heat stress in *Mytilus* mussels and differences in the abundance and activity of apoptotic proteins appear to contribute to interspecific differences in temperature tolerance [82]. Greater numbers of hydrogen bonds and salt bridges within apoptotic proteins could influence temperature tolerance of *Mytilus* mussels by allowing removal of irreparable cells to continue in *M. galloprovincialis* beyond temperatures that had caused proteins within cell death pathways to become non-functional in *M. trossulus*. *M. galloprovincialis* proteins that could exert a strong influence on cell fate during heat stress, including p63, DNA damage regulated protein (PDRP), and multiple caspase enzymes [72, 73, 83], had more hydrogen bonds and salt bridges per amino acid than *M. trossulus* proteins or other functional classes of *M. galloprovincialis* proteins. p63 is a member of the p53 family of transcription factors that serve as connection points for several stress response pathways and that govern the decision between cell survival and apoptosis by controlling the abundance of gene products that determine cell fate [70, 71, 73, 84, 85]. PDRP is a downstream target of p53, suggesting that this molecule also contributes to the balance between cell survival and death during environmental stress [73, 86]. Caspases are peptidases that degrade diverse substrates as part of apoptosis [72, 87, 88], and activity of these enzymes is necessary for removal of damaged macromolecules during heat stress. Caspases are also dependent on a large conformational change to convert from an inactive to active form [89], which is consistent with structural changes associated with temperature adaptation occurring disproportionately in proteins with this characteristic.

### Differences in amino acid use among *Mytilus* proteins

Prokaryotes living in extreme environments exhibit signatures of temperature adaptation in the amino acid composition of their proteins [6, 7, 9]. For proteins adapted to extreme heat, this imprint includes a higher proportion of hydrophobic, charged, and large volume residues, as well as an increase in the number of cysteines and prolines, and a decrease in glycine content [1, 3, 4]. Differences in amino acid use between *M. galloprovincialis* and *M. trossulus* indicate that some, but not all, of these adaptive mechanisms contribute to thermal adaptation in *Mytilus* proteins. Selection for protein function under considerably different temperature regimes is a plausible explanation for inconsistencies in amino acid use between *Mytilus* and prokaryotic extremophiles. Changes in protein stability that underlie small differences in habitat temperature and organism thermal tolerance, such as that occurring between *M. galloprovincialis* and *M. trossulus*, may favor particular mechanisms of structural change or use mechanisms observed in extremophiles much less frequently.

Increasing stability via addition of hydrogen bonds appears to be a method of high temperature adaptation used in proteins of both *Mytilus* mussels and thermophilic prokaryotes. Hydrogen bonds are found in greater abundance in thermophile proteins [32, 33], are less abundant in psychrophilic proteins [41–43], and consistent with this pattern, the upper 25 and 50% of *M. galloprovincialis* proteins with the most hydrogen bonds per amino acid had significantly more hydrogen bonds than did the upper 25 and 50% of *M. trossulus* proteins with the most hydrogen bonds per amino acid. Relative increases in hydrogen bonding among *M. galloprovincialis* proteins were accompanied by a net increase in amino acids most likely to form hydrogen bonds. Threonine, asparagine, and glutamine exhibited large proportional increases in *M. galloprovincialis* proteins relative to *M. trossulus* proteins, and all three of these residues have both donor and acceptor atoms in their side chains that facilitate formation of hydrogen bonds [90].

Increasing the number of salt bridges within proteins represents another mechanism of high temperature adaptation shared by *Mytilus* and thermophile proteins. The stabilizing effect of salt bridges has long been implicated in the evolution of heat tolerant proteins [91–94], and the upper 25 and 50% of *M. galloprovincialis* proteins had significantly more salt bridges per amino acid than did the upper 25 and 50% of *M. trossulus* proteins. Because salt bridges are the result of electrostatic interaction between ionized groups of opposite charge [95], the relative increase in salt bridges among *M. galloprovincialis* proteins was expected to have occurred through substitution of charged for uncharged amino acids. A greater proportion of charged residues is one of the most frequently observed characteristics of thermophilic proteins [7, 13–17] and temperature-related differences in the function of IDH orthologs between *Mytilus* congeners were attributed to insertion of charged residues [46]. However, the indication from analysis of larger numbers of *Mytilus* proteins is that temperature adaptation in these species is not as simple as adding charged residues. Contrary to expectations, there was an overall decrease in the proportion of charged amino acids in the upper 25 and 50% of *M. galloprovincialis* proteins with the most salt bridges per amino acid relative to *M. trossulus*. Opposing directions of change between positively and negatively charged residues may explain this counterintuitive result. The overall decrease in charged amino acids among *M. galloprovincialis* proteins was principally caused by large declines in positively charged lysine and arginine; in contrast, the total number of negatively charged aspartate and glutamate increased in *M. galloprovincialis* proteins relative to *M. trossulus* proteins. Despite the decrease in lysine use in *M. galloprovincialis* relative to *M. trossulus*, lysine content remained high among the proteins of both species when compared with other amino acids: 6.7% of all residues in the upper 50% of *M. galloprovincialis* proteins with the most salt bridges per amino acid were lysine (fourth highest use rate) and 8.7% of residues in *M. trossulus* proteins with the most salt bridges per amino acid were lysine (highest use rate) (Table 5). Given that lysine outnumbered most other amino acids in these sets of *Mytilus* proteins, it is probable that most positively charged residues were not interacting with a negatively charged residue to form a salt bridge. Heat adaptation in *M. galloprovincialis* may have therefore involved an increase in the proportion of negatively charged amino acids so that new electrostatic interactions could form with the excess lysines already present, ultimately resulting in new salt bridges and more stable proteins compared with *M. trossulus*.

*Mytilus* congeners did not appear to modulate either hydrophobicity or volume in a majority of the proteins analyzed here, revealing a potential dichotomy between mechanisms of protein temperature adaptation used by extremophiles and those used by *Mytilus*. Proteins adapted to extreme heat have been repeatedly shown to increase the content of non-polar, hydrophobic amino acids at the expense of polar, hydrophilic residues such as serine, threonine, asparagine, and glutamine [6, 13, 18]. One analysis showed that thermophilic proteins preferred nearly every other amino acid to uncharged, polar residues, with removal of serine, threonine, asparagine, or glutamine accounting for 52 of 64 possible replacements in the proteins analyzed [6]. This pattern of substitution resulted in a net loss of nine uncharged polar residues in a typical 300 amino acid thermophilic protein [6]. Contrary to these trends, the content of polar, more hydrophilic amino acids increased in *M. galloprovincialis* proteins compared with proteins of *M. trossulus*. The increase in polar amino acids is presumed to have occurred as a means of enhancing stability through additional hydrogen bonding, rather than increasing hydrophobicity, a premise that has support in *Mytilus*. The single amino acid substitution responsible for differences in the Km-temperature relationship of cMDH between *M. galloprovincialis* and *M. trossulus* was the result of a non-polar (valine) to polar (asparagine) substitution and formation of an additional hydrogen bond [45]. There was also significant positive correlation between the content of polar residues and organism temperature tolerance among cMDH orthologs of marine mollusks that included both *Mytilus* species [12].

Contrasting trends of amino acid use between *Mytilus* and extremophiles suggests that different mechanisms of evolution are at work when proteins adapt to moderate temperatures than when proteins adapt to temperature extremes [12]. Maximizing the hydrophobic effect may be required for the evolution of exceptionally stable proteins that function at the highest end of temperatures found in the biosphere. However, hydrophobicity may be less important when proteins adapt to more modest heat stress. In support of this conjecture, the hydrophobic effect makes a larger contribution to protein stability than does hydrogen bonding [96–98]. Furthermore, the upper thermal limit of protein function may not be the factor directly under selective pressure in *Mytilus* mussels, as the upper critical temperatures of these species is likely lower than the denaturation temperature of most of their proteins [5, 45, 46, 99]. Instead, selection may be acting to make small adjustments in local flexibility such that critical aspects of protein function, such as substrate binding or catalytic rate, are optimized at different temperatures [5, 46]. Changes in hydrogen bonding may be all that is required for small shifts in the optimal operating temperatures of *M. galloprovincialis* and *M. trossulus* proteins.

Shifts in the abundance of individual amino acids have also been correlated with increases in protein thermal stability and enhanced function at high temperatures [3, 4, 100]. Increasing the number of cysteines can enhance protein stability through the formation of disulfide bonds between sulfur atoms on adjacent residues [101, 102]. There were 109 more cysteine residues than expected in all *M. galloprovincialis* proteins, implying that additional disulfide bonds contribute to greater protein thermal stability and organism temperature tolerance in this species compared with *M. trossulus*. Disulfide bond formation generally occurs within the endoplasmic reticulum, and consequently, potentially adaptive shifts in disulfide bonding may be predominantly occurring in a smaller subset of extracellular *Mytilus* proteins [103]. Glycine content tends to decrease in thermophilic proteins because the absence of a sidechain in this residue imparts greater conformational flexibility that is assumed to be selected against at high temperatures [4, 100]. The increase in glycine content of *M. galloprovincialis* proteins compared with *M. trossulus* proteins (39 more residues than expected) suggests this adaptive mechanism does not affect the majority of proteins analyzed here. Glycine use in *Mytilus* more closely resembles that observed among mollusk orthologs of cMDH, where glycine content was positively correlated with organism temperature tolerance. In this case, addition of glycine to cMDH of heat-tolerant mollusks was theorized to organize the folded protein into a more compact and temperature-resistant structure [12]. Thermal stability of mollusk cMDH orthologs was also positively correlated with total proline content, which was predicted to slow heat denaturation by stabilizing sites of contact between subunits of the enzyme [12]. *M. galloprovincialis* proteins did not contain a higher than expected number of proline residues compared with *M. trossulus* proteins. Differences in proline content between *Mytilus* and other mollusk species may be another indication that certain types of adaptive change are favored at different magnitudes of temperature stress. The strongest evidence for the functional importance of proline in mollusk cMDH resulted from comparison of two snails (*Echinolittorina malaccana* and *Littorina keenae*) that have much higher temperature tolerances (LT_50_ = 57 °C and LT_50_ = 48 °C, respectively) than either *M. galloprovincialis* or *M. trossulus* (LT_50_ = 38 °C and LT_50_ = 34 °C, respectively) [12].

### Caveats and future directions

Conclusions about the evolutionary influence of temperature on the structure of *Mytilus* proteins should be considered in the context of several caveats. A primary motivation for this study was to analyze potentially adaptive change over a very large number of proteins. The methodology used here was successful in collecting data from a sufficient number of proteins to provide insight into the proportion of the proteome modified during temperature adaptation, detect functional bias among proteins acted on by natural selection during temperature adaptation, and identify mechanisms of protein temperature adaption that are conserved or divergent across phylogeny. However, disparity in protein sequence resources between congeners, lack of homology to proteins with solved three-dimensional structures, and duplicate sequences in the UniProt database, reduced the number of *Mytilus* proteins that could be analyzed. While a far greater number of proteins were evaluated in this study compared with most previous work, it is possible that the 58 *M. trossulus* proteins did not represent the full variation of hydrogen bonding, salt bridge formation, or amino acid use found across the proteome of this species. Differences in the number of hydrogen bonds and salt bridges between *Mytilus* proteins could therefore have resulted from omission of certain *M. trossulus* proteins rather than adaptive change in *M. galloprovincialis*. Sequencing the genomes of these species would increase the number of proteins that could be compared and allow this uncertainty to be empirically tested [104].

The methodology used here identified differences in the total number of hydrogen bonds, salt bridges and amino acids among *Mytilus* proteins, but did not include an evaluation of the location of adaptive change within proteins. Structural changes occurring in specific regions of *Mytilus* proteins could have greater impacts on thermal sensitivity than change occurring elsewhere in the protein. Amino acid substitutions that influence the stability of mobile regions, regions of subunit interaction, or coiled-coil domains, have all been hypothesized as important for temperature adaptation of *Mytilus* proteins [5, 12, 53]. Adaptive amino acid substitutions occurring among orthologs of dehydrogenase enzymes from marine organisms adapted to different temperatures tend to occur on the solvent exposed surface, rather than other regions of the protein [5]. Furthermore, adaptation through adjustments in local-scale protein stability can occur without a concomitant change in global protein stability [46, 99, 105–107], suggesting that it is possible for adaptive change to occur without altering the total number of hydrogen bonds or salt bridges or without obvious deviations in amino acid composition. Development of high-throughput approaches that can distinguish stabilizing interactions in specific regions of proteins (e.g. surface vs. core) is necessary to identify hotspots of evolutionary change.

The ability of in silico modeling algorithms to accurately capture subtle changes in protein structure has been questioned [5, 12, 108]. Single amino acid substitutions have been repeatedly shown to underlie structural and functional differences in the enzymes of marine ectotherms, including between *Mytilus* mussels [8, 45, 109, 110]. Orthologs of IDH differ by only two non-conservative amino acid substitutions between *M. galloprovincialis* and *M. trossulus*, and orthologs of cMDH differ by only a single non-conservative change between the two species [45, 46]. The methodology used here predicted a small increase in the total number of hydrogen bonds in *M. galloprovincialis* orthologs of IDH and cMDH compared with orthologs of *M. trossulus*, a result that is consistent with small differences in the amino acid sequences of these orthologs and the temperature tolerances of each species. However, comparison of structure-function relationships among other mollusk cMDH shows that in silico predictions of protein stability do not always replicate in vitro measures of thermal performance [5, 12]. High-throughput experiments that couple in silico with in vitro or in vivo analyses may address this problem, but will require development of new methods capable of assessing larger numbers of proteins in vitro or in vivo.

The presence or absence of ligands in *Mytilus* protein models could also influence patterns of hydrogen bonding described in this study. The HBOND algorithm used here considers the atomic distance of all potential donor and acceptor atoms listed in the .pdb file, including those present in the ligand [60]. A *Mytilus* protein model with the ligand included may therefore have a different number of hydrogen bonds compared with a model of the same protein without the ligand. The methodology used here prioritized selection of the most accurate template and did not account for the presence or absence of ligands in the chosen template. This approach was chosen to maximize the number of proteins that could be analyzed, rather than restrict analysis to a much smaller subset of proteins with or without ligands. Future analyses may allow for the effect of ligands on hydrogen bonding and salt bridge formation to be explicitly quantified if more protein sequences are obtained for *M. galloprovincialis* and *M. trossulus*.

A final consideration is that *Mytilus* species compared here also differ in their tolerance of salinity: *M. galloprovincialis* is more tolerant of increases in salinity and *M. trossulus* is more tolerant of decreases in salinity [44, 50]. Some of the protein structural changes examined here could therefore reflect selection pressure resulting from differences in habitat salinity rather than temperature. The proteins of salt-tolerant halophiles can be enriched with acidic amino acids, such as aspartate and glutamate, because side-chains from these negatively charged residues compete with cations in the environment and prevent protein aggregation through electrostatic repulsion [111]. The proportion of aspartate and glutamate was higher in more salt-tolerant *M. galloprovincialis* proteins than in less salt-tolerant *M. trossulus* proteins. Additional experiments are required to separate proteins contributing to thermal tolerance from those that underlie salinity tolerance in *Mytilus*.

## Conclusions

Comparison of large numbers of proteins between warm-adapted *M. galloprovincialis* and cold-adapted *M. trossulus* indicates that protein structures from these two species vary in a manner that is consistent with adaptation to different temperatures, but that does not always conform to mechanisms of temperature adaptation observed in extremophiles. Only a portion of the proteins examined exhibited significant differences in the number of hydrogen bonds and salt bridges between *Mytilus* congeners, implying that comprehensive modification of the proteome is not required for small shifts in temperature tolerance and that adaptive responses to climate warming may be feasible in these species [112]. Enzymes, in particular those involved in oxidative stress responses and programmed cell death, were over-represented among proteins with the greatest number of hydrogen bonds and salt bridges in more heat tolerant *M. galloprovincialis*. This result is indicative of bias during temperature adaptation toward proteins in which function is dependent on large conformational change [46, 99, 107]. Structural changes in *Mytilus* proteins were associated with shifts in amino acid use that were likely to alter the number of hydrogen bonds, salt bridges, and disulfide bonds, but unlikely to substantially change hydrophobicity, volume, or proline and glycine content. Evolutionary changes required to maintain protein function in response to small shifts in environmental temperature are therefore likely to differ from those needed for function at temperature extremes.

## Supplementary information

**Additional file 1: Figure S1.** All unique proteins QMEAN >-5 *M. galloprovincialis*. **Figure S2.** All unique proteins QMEAN >-5 *M. trossulus*. **Figure S3.** All unique orthologs QMEAN >-5. **Figure S4.** Over-representation of *M. galloprovincialis* protein ontologies. **Figure S5.** Spearman rank correlation between the number of hydrogen bonds and salt bridges per amino acid among *Mytilus* proteins. **Figure S6.** Amino acid composition all unique proteins *M. galloprovincialis*. **Figure S7.** Amino acid composition all unique proteins *M. trossulus*. **Figure S8.** Chi square tests of independence comparing amino acid use in different sets of *Mytilus* proteins. **Figure S9.** Spearman rank order correlation between residue hydrophobicity or residue volume and deviation in amino acid use in *M. galloprovincialis* relative to *M. trossulus* for different sets of proteins.

## Data Availability

The datasets supporting the conclusions of this article are included within the article and its additional files. Code used to create the software for quantification hydrogen bonds, salt bridges, and amino acids and R scripts used for TopGO over-representation analysis have been deposited in GitHub (user: tylergevans; repository: Mytilus-Protein-Adaptation).

## Abbreviations

ATP: Adenosine triphosphate
cMDH: Cytosolic malate dehydrogenase
DNA: Deoxyribonucleic acid
ESBRI: Evaluating Salt BRIdges in proteins
IDH: Isocitrate dehydrogenase
Km: Michaelis-Menten constant
LT_50_: Lethal temperature for 50% of tested organisms
NADH: Nicotinamide adenine dinucleotide
NADPH: Nicotinamide adenine dinucleotide phosphate
pdb: Protein database
PDRP: P53 and DNA damage regulated protein
QMEAN: Qualitative Model Energy ANalysis
ROS: Reactive oxygen species
